# Longitudinal associations between psychological factors, community integration, and suicide risks among patients with treatment-resistant depression in Vietnam

**DOI:** 10.3389/fpsyt.2025.1597196

**Published:** 2025-07-28

**Authors:** Pham Thi Thu Huong, Chia-Yi Wu, Lee Ming Been, Nguyen Van Tuan, Pham Thi Thu Hien, Nguyen Thi Lan Anh, Nguyen Thi Son, Nguyen Thi Thu Hien

**Affiliations:** ^1^ Faculty of Nursing and Midwifery, Hanoi Medical University, Hanoi, Vietnam; ^2^ School of Nursing, College of Medicine, National Taiwan University, Taipei, Taiwan; ^3^ Department of Nursing, National Taiwan University Hospital, Taipei, Taiwan; ^4^ Taiwan Association Against Depression, Taipei, Taiwan; ^5^ Taiwanese Society of Suicidology and Taiwan Suicide Prevention Center, Taipei, Taiwan; ^6^ Department of Psychiatry, Shin-Kon Wu Ho Su Memorial Hospital, Taipei, Taiwan; ^7^ National Institute of Mental Health, Bach Mai Hospital, Hanoi, Vietnam; ^8^ Department of Psychiatry, Hanoi Medical University, Hanoi, Vietnam; ^9^ Department of Nursing, Hanoi Medical University Hospital, Hanoi, Vietnam; ^10^ Department of Dermatology and Burns, Bach Mai Hospital, Hanoi, Vietnam

**Keywords:** treatment-resistant depression, longitudinal study, community integration, suicide ideation, suicide attempt, psychological fluctuation

## Abstract

**Objective:**

To examine longitudinal associations of psychological distress, community integration, suicide ideation, and attempts within 3-month post-discharge period among patients with treatment-resistant depression (TRD).

**Methods:**

Prospective study design with a 3-month followed-up from the hospital admission through community reintegration. All the 53 patients with TRD were interviewed one week after admission (T0) plus 1-month (T1) and three months (T2) after discharge with a structured questionnaire from October 2021 to September 2022. Descriptive and correlational analysis of the trend and associates of TRD suicide risks were performed.

**Results:**

The results revealed that suicide ideation and attempt rates reduced after inpatient treatment, but increased altogether from T1 to T2 in the community. Nearly half of the participants (46%) reported recent suicide ideation, and 13.46% attempted suicide at T2. Perceiving poor quality of life and a low level of community integration performance were associated modestly with suicide ideation, with 1.02- and 1.10-times higher risk, respectively. On the other hand, a high level of psychological distress increased the hazard of suicide attempts by 1.13-fold.

**Conclusions:**

Our findings suggest future suicide prevention strategies and the importance of regular assessment of inpatients and outpatients for psychological distress to identify and engage high-risk individuals.

## 1 Introduction

Globally, major depressive disorder (MDD) is the third leading cause of years lived with disability (1) and accounts for the highest proportion of disability-adjusted life years, with 5.55 times higher in low and middle-income countries than high-income countries (2). Moreover, MDD is the primary cause of disability for chronically ill patients living in the community and a major risk for suicide (3, 4). The number of individuals with depression is approximately 350 million people worldwide, of that 76-87% of people in low-and middle-income countries receive no treatment (5). According to Vietnamese national data sources, the prevalence of mental health disorders was 14.2%, with 2.45% depressive disorders, and the crude suicide rate was 7.5/100.000 (6). The goals of MDD treatment are to control symptoms, achieve remission and assist patients in recovery functioning level. However, as many as 50% of patients who received first-line depression treatment did not achieve remission (7). The most commonly used definition of TRD for MDD was based on the failure of response to at least two treatments of an adequate dose and duration, with the minimum duration cited as four weeks typically (8). Despite the varied definition across the world, two consensuses among the Asian population defined the TRD as failure of at least two antidepressant trials from the same or different classes at an adequate dose for 6–8 weeks during any MDD episode (9, 10). Up to date, studies of TRD were limited in clinical and research settings in Asian countries. Adopting a universal definition for TRD is necessary to reduce the heterogeneity and misclassification of TRD patients, which may assist future policymaking or clinical services.

TRD patients are twice as likely to be hospitalized with higher medical costs, and healthcare resource utilization identified compared to those without TRD (11, 12). These direct and indirect medical costs among TRD patients due to the need for additional treatment led to increased use of medical resources and work and activity impairment (13, 14). In addition, the finding addressed these economic burdens and poor quality of life attributed to TRD in a depressive episode (14). The impact and burden of TRD are immense and go far beyond their economic cost; that is often not only associated with increased suicidality but also mortality (15–17). A growing body of research has supported that individuals with TRD have a significantly increased risk of all-cause mortality (18, 19), high prevalence of suicide attempts, and suicide ideation (15, 20, 21).

Evidence revealed that the highest suicide risk among those hospitalized for affective disorders was after three months of discharge. A recent study in the United States showed that patients with depression were the most hazardous group of completed suicide within 3-month post-discharge compared to other depressive inpatients, with 16.6 times substantially higher than the general population (22). Similarly, a recent meta-analysis of 100 studies found that the suicide rate was highest within 3-month after discharge and 4-fold higher among those admitted with suicide ideation or attempt (23). Despite this, post-discharge studies of patients with TRD were scarce with suicide rate outcomes. Only 13.2% of the general population who attempted suicide in Vietnam had psychiatric treatment, and up to 52.5% had no treatment or medical contact in the community (24). Similarly, another study conducted at the Poison Center of Bach Mai hospital indicated that 92% of suicide attempters had no contact with psychiatric services before their behaviors (25). In clinical observation, TRD patients with high suicide risk admitted to the National Institute of Mental Health (NIMH) for treatment tend to have different characteristics and diagnosis composition in Vietnam. Therefore, patients with TRD need to be investigated during hospitalization and short-term post-discharge. Meanwhile, it is critical to developing strategies for suicide prevention and depression management.

Compared to the general public, TRD patients appeared more severe in psychological distress by 7.2-fold (20, 26). Further, the hazards for completed suicide among moderate or severe psychological distress were 1.37-fold and 4.16-fold, respectively (27). This evidence suggested that chronic psychological distress was a serious concern that requires more attention in this TRD group and their family members. Therefore, the challenges of long-term functional impairment, high recurrence, and prominent suicidal risks in TRD require a holistic approach with adequate resources in hospitals and community settings (28). A recent study indicated that during the trajectory of community recovery, patients with TRD faced difficulties of community life reintegration (i.e., home, social, and productivity integration), which were associated with recent suicide ideation and overall suicide risk (20). Indeed, well-integrated life in the community was associated with a reduction in current suicide ideation (29) and was an essential concept that may affect an individual’s recovery level among depressive patients (26). However, to design appropriate strategies, the correlation between life integration and suicide risks needs to be further investigated in longitudinal study designs.

Vietnamese mental health care system focuses mainly on both provincial and national levels, providing minimal quantity and care services to follow-up patients in the community (30). Hence, it may explain the higher prevalence of inpatient depression treatment in Vietnam compared to developed countries. Community mental health networks in Vietnam have mainly focused on screening, prescription, and monthly follow-up for medication only that covers patients with schizophrenia and chronic epilepsy since 1999 (31). Depression has received limited attention from policymakers since 2015 but faces many difficulties such as limited mental health community facilities, lack of staff with mental health backgrounds in primary healthcare, and government resources distribution for depression care networks (31). Similarly, a recent study showed the Vietnamese mental health care system’s limitations, including inappropriate mental health policy and service organization, shortage of human resources, and lack of evidence-based interventions (32). Further study of the clinical and suicide risk of TRD, especially among patients hospitalized for an episode of MDD, is warranted to characterize better factors associated with suicidality for future mental health service system.

## 2 Methods

### 2.1 Study design and data collection

This was a longitudinal study with 3-month followed-up from hospital to the community. Firstly, the first author performed quantitative investigations in the study hospital to collect inpatient baseline information. The hospital is the largest and leading comprehensive general hospital in Vietnam, with a total of 3500 beds, 4500 personnel, and 56 departments and institutes. NIMH has 258 beds with approximately 4000 inpatients and 70.000 out-patients per year. Most patients come from Hanoi and other provinces from Central to North Vietnam.

**Figure 1:**
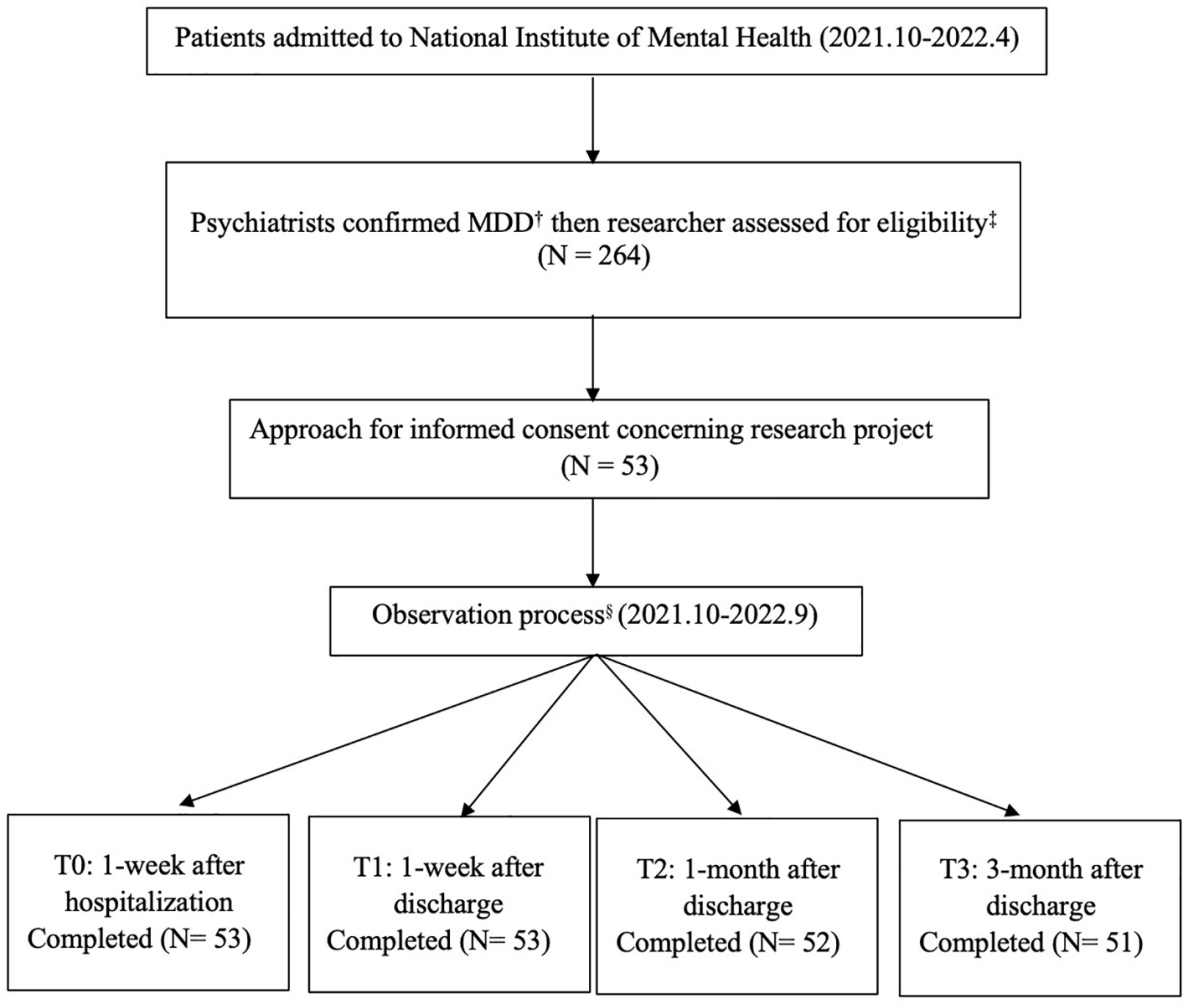
Data collection procedure. ^†^MDD, Major depressive disorder; ^‡^Screening and recruitment from 1^st^ October 2021 to 10^th^ April 2022; ^§^Observation process from 1^st^ October 2021 to 3^rd^ September 2022.

Data were collected from patients diagnosed with MDD by primary psychiatrists and admitted for inpatient treatment from 1^st^ October 2021 to 10^th^ April 2022 (**Figure 1**). During the first week of admission, the study aims and procedures were explained to the patients by the first author to seek their consent to participate; meanwhile, the medical records were reviewed to confirm eligibility. Finally, written consent was obtained from all the participants. The participants were interviewed for the first time after 1-week hospitalization (T0) and completed the interview after discharge 1-month (T1) and 3-month (T2) at the out-patient department or via telephone if not coming back for out-patient appointments. Of the 264 inpatients diagnosed with MDD during the screening period, 53 patients were found to be eligible for TRD (20.08% of all MDD patients). Of the 53 patients, 52 completed the interview after 1-month discharge, 51 completed the interview after 3-month discharge. The drop-out rate was 3.8% (n=2). All the participants underwent regular available treatment (treatment as usual) during the observation. The authors adhered to the Reporting of Observational Studies in Epidemiology (STROBE) Statement in **Supplementary File S1** (https://www.equator-network.org).

### 2.2 Participants

In the present study, we recruited patients who met the diagnoses of major depression by treating psychiatrists according to the International Classification of Disease – Tenth revision, clinical modification (ICD-10 F32: Major depressive disorder, single episode, F33: major depressive disorder, recurrent). TRD is defined as MDD with failure of at least two antidepressant trials from the same or different classes at an adequate dose for 6–8 weeks during any MDD episode (9, 10). However, fifty-eight percent of Asian physicians would treat their patients for 4–8 weeks before determining antidepressant failure (9), so we used a duration of at least 4–6 weeks in our criteria. We applied the Maudsley Staging Method (MSM) (33) to assess the duration of illness, symptom severity (classified through the fourth position in ICD-10 diagnoses of MDD), levels of treatment-refractory with a history of antidepressants failures or augmentation. The research team applied the defined threshold of TRD with a score of ≥5 in MSM to capture the TRD patients (34). The researcher also screened in those fit our TRD criteria by using two extra questions, “Have you ever experienced using two kinds of antidepressants continuously for at least 4–6 weeks?” and “Do you perceived your recovery in depressive symptoms to be less than 50%?”. All the 53 patients in our sample answered “yes” to these questions. We excluded patients with a history of psychosis, bipolar or related disorders, such as intellectual disability, severe alcohol or other substance use disorder, organic brain damage, dementia, and detectable neuro-cognitive impairment.

### 2.3 Measures

#### 2.3.1 Demographic characteristics

Information collected included gender, age, education level, relationship status, working status, religious belief, household income in recent years, duration of having depression and length of hospitalization, current diagnosis.

#### 2.3.2 Psychological variables

##### 2.3.2.1 The 5-item brief symptoms rating scale

The BSRS-5 is a self-report questionnaire developed from the Symptom Checklist-90-Revised (SCL-90R) and 50-item Brief Symptom Rating Scale (BSRS-50) to measure the level of psychological distress (35). The participants were asked to answer the survey containing five psychopathological symptoms in the past week: insomnia, anxiety, hostility, depression, and inferiority. An additional item was inquired to assess recent suicide risk, “Do you have any suicide ideation in the past 7 days including today?”. The participants rated the symptoms on a 5-point Likert scale from 0 (not at all) to 4 (extremely). A total score was calculated by computing the first five items. The cut-off points of 5/6, 9/10, and 14/15 stand for mild/moderate/severe levels of psychological distress or psychiatric morbidity. The BSRS-5 showed moderately good predictive validity in community out-patients and daycare patients (Cronbach alpha 0.79 and 0.80, respectively). The BSRS-5 Vietnamese version in this study also demonstrated adequate internal consistency with Cronbach alpha=0.78.

##### 2.3.2.2 Quality of life (EQ-VAS)

The EQ-5D-5L which includes the visual analog scale (EQ-VAS) and a 5-dimensions questionnaire was introduced in Vietnam in 2012 with a wide range of target populations and validated by the nationally representative sample in 2020 (36). We used the EQ-VAS score to interpret whether the person had a good life quality. This one-item measurement assesses perceived quality of life by asking about their current states of wellness ranging from 0 to 100, separating “the worst health you can imagine” and “the best health you can imagine” (37).

#### 2.3.3 Suicide risk assessment

*Suicide ideation*: We inquired recent suicide ideation, which means whether the patient had any suicide ideation over the past week and including today, which is included in the 6th item of the BSRS-5. The answers were categorized as “Yes=1” with a score from 1 to 4 and “No=0”.

*Suicide attempt*: The patients were asked “Have you ever tried to hurt yourself/attempted suicide during the past month?”. The answers were categorized as “Yes=1” or “No=0”.

*Other suicide related information*: We also assessed if the patients had lifetime suicide ideation, lifetime suicide attempt/self-harm, and family suicide history.

#### 2.3.4 The revised community integration questionnaire

The original version of the CIQ with 15-item (38) has been translated into a dozen different languages and widely utilized to assess the outcomes of rehabilitation following traumatic brain injury, stroke, spinal cord injury, developmental disabilities, and a mental illness which provided further direction for clinical or research settings. The revised version of the CIQ (CIQ-R) has been expanded to include three questions relating to experience of using cell phones and social media for community integration (39). The 18-item CIQ-R assessed four aspects of life: home integration, social integration, productivity, and electronic social networking. The total score of CIQ-R ranges from 0 to 35, with higher scores indicating better integration level or social activity involvement in community settings. The Vietnamese version of CIQ-R has demonstrated adequate internal consistency with Cronbach alpha = 0.79 in the present study.

### 2.4 Data analysis

Statistical calculations were carried out using SPSS 25.0 (Chicago, IL). In this follow-up study, only two patients lost follow-up (one female and one male); the first author could not contact them after five calls and several messages. The last patient, ID53, was waiting for the final interview after 3-month discharge. After confirming data completeness for analyses, descriptive analysis was performed for each variable to check rationality. The means, standard deviations, and frequencies were calculated for quantitative variables. In addition, continuous variables such as psychological variables, including psychological distress (BSRS-5), quality of life (EQ-VAS), and community integration (CIQ-R), underwent descriptive analysis normality plotting to check outliers and their suitability in association statistics. No outlier value was detected in box-plots of all these key variables. Cronbach alpha was used to estimate the internal consistency of BSRS-5 and CIQ-R.

To find the significance between time factors and within factors among key variables such as psychological distress (BSRS-5), quality of life (EQ-VAS), community integration (CIQ-R), suicide ideation in recent one week, and suicide attempt in recent month during 3-wave interviews, we performed analysis using One-way ANOVA in the form of fixed-effect regression. In addition, we created dummy variables reflecting patients and three-time points. Further, we applied the univariable Cox regression analyses to examine a wide range of possible predictors (demographic, psychological distress, quality of life, and community integration) for suicide ideation and attempt. Finally, a stepwise multivariate Cox regression model was used to identify factors associated with suicide ideation and attempt during the follow-up period. Multicollinearity was checked by evaluating the VIF scores and found no multicollinearity problems with all VIF score less than 3.3 (40). Findings were considered statistically significant if the p-value < 0.05.

### 2.5 Ethical considerations

The study adhered to the Declaration of Helsinki with ethical approval provided by the Institutional Review Board at Hanoi Medical University (reference number: IRB00003121). All patients were provided informed consent and were informed that they were free to withdraw from the study at any time. Suicide assessment is a sensitive issue in Vietnamese culture, so the interviews were only conducted in a private safe place when the patients felt able and willing to share their feelings. All patients were provided a safety plan that included recognition of warning signs, seeking resources for crisis management, and a plan to restrict access to lethal means. Participants could also contact the interviewer during significant events (severe suicide ideation or attempt). Hence, seven patients with a suicide attempt and others with severe suicide ideation had contacted the first author for emotional support and were admitted to the hospital again or visited psychiatric clinics shortly thereafter. Confidentiality was maintained by using codes to identify participants.

## 3 Results

As shown in **Table 1**, there were 53 patients who participated in this longitudinal study; two-thirds were female (71.7%), and the average age was 48 ± 12.95 years. One-third of patients had a vocation school or college-level (35.8%). Of the participants, 41 (77.40%) were married or living with a partner; three-quarters (77.36%) had a full-time, part-time job or studied while admitted to the hospital. More than half of patients (52.8%) had a religious belief or frequently visited the church or pagoda. Sixty-two percent of the participants reported a household income of less than ten million VND (US$ 435). The average duration of depression and length of hospitalization was 56.64 ± 61.19 months and 20.81 ± 11.23 days, respectively. Most patients were diagnosed with F33 (71.6%) and 28.4% with F32 according to ICD-10 criteria. Almost patients had lifetime suicide ideation (98.1%), but only nearly half (45.3%) attempted suicide during their lifetime, and only five participants (9.4%) reported a family suicide history. **Table 1** also shows the results of univariable Cox regression analyses of suicide predictors. In these analyses, only the demographic characteristic of no religious belief was associated with a higher risk for suicide ideation; younger age, staying single/divorce/separate/widow, and having a history of lifetime suicide attempts were significantly correlated with a higher risk for suicide attempts.

**Table 1:** Characteristics of the participants at baseline and their correlations with suicide ideation and attempt during the 3-month follow-up (N = 53).

Characteristics	Mean ± SD n (%)	Suicide ideation^†^ (past 1 week)		Suicide attempt^†^ (past 1 month)	
		B (SE)	HR (95% CI)	B (SE)	HR (95% CI)
Gender					
Female	38 (71.70)	1		1	
Male	15 (28.30)	–0.08 (0.24)	0.93 (0.57 – 1.49)	–0.29 (0.51)	1.75 (0.28 – 2.02)
Age	48.40 ± 12.95	–0.00 (0.01)	0.99 (0.98 – 1.02)	–0.06 (0.02)	0.94 (0.91 – 0.97)***
Education level					
Primary school	3 (5.70)	1		1	
Secondary school	23 (43.40)	0.00 (0.48)	1.00 (0.39 – 2.55)	0.66 (0.44)	1.95 (0.83 – 4.58)
High school		–0.03 (0.53)	0.98 (0.35 – 2.74)	0.04 (0.40)	0.95 (0.33 – 2.52)
Vocation school and College	19 (35.80)	0.04 (0.48)	1.04 (0.41 – 2.67)	0.03 (0.40)	1.01 (0.28 – 2.55)
Relationship status					
Single/divorce/separate/widow	12 (22.60)	1		1	
Married/lived with partner	41 (77.40)	–0.11 (0.25)	0.89 (0.55 – 1.45)	–1.18 (0.43)	0.31 (0.13 – 0.71)**
Working status					
No (Unemployed/retired)	12 (22.64)	1		1	
Yes (Full-time/part-time job/student)	41 (77.36)	–0.18 (0.22)	0.84 (0.54 – 1.29)	0.39 (0.43)	1.47 (0.64 – 3.40)
Religion belief					
No	25 (47.20)	1		1	
Yes	28 (52.80)	–0.43 (0.22)	0.65 (0.43 – 0.99)*	0.57 (0.46)	1.77 (0.72 – 4.35)
Household income (1usd = 23.000 VND)					
< 10 million VND (US$ 435)	33 (62.30)	1		1	
≥ 10 million VND (US$ 435)	20 (37.70)	–0.26 (0.23)	0.77 (0.49 – 1.22)	0.71 (0.43)	2.03 (0.88 – 4.71)
Time has depression (month)	56.64 ± 61.19	0.00 (0.00)	1.00 (0.99 – 1.00)	0.01 (0.01)	1.00 (0.99 – 1.01)
Length of hospitalization (day)	20.81 ± 11.23	0.01 (0.01)	1.01 (0.99 – 1.03)	–0.04 (0.03)	0.96 (0.92 – 1.01)
Current diagnosis (ICD-10)					
F32.2	15 (28.30)	1		1	
F33.1	1 (1.90)	–0.17 (1.02)	0.84 (0.11 – 6.25)	1.37 (1.02)	0.02 (0.01 – 0.03)
F33.2	29 (54.70)	0.02 (0.25)	1.02 (0.62 – 1.67)	0.85 (0.64)	2.35 (0.67 – 8.24)
F33.3	8 (15.10)	0.15 (0.32)	1.17 (0.62 – 2.20)	1.07 (0.73)	2.92 (0.69 – 12.24)
Lifetime suicide ideation (yes)	52 (98.10)	3.03 (3.47)	8.69 (1.27 – 20.69)	3.03 (3.87)	8.99 (2.20 - 18.26)
Lifetime suicide attempt/self-harm (yes)	24 (45.30)	–0.27 (0.23)	0.77 (0.49 – 1.20)	2.21 (0.62)	9.10 (2.69 – 30.77)***
Family suicide history					
No	48 (90.60)	1		1	
Yes (01 still alive; 04 death)	5 (9.40)	1.92 (0.33)	1.21 (0.63 – 2.34)	0.78 (0.56)	2.17 (0.73 – 6.46)

*p <.05, **p <.01, ***p <.001; ^†^Based on univariable Cox regression analyses.

As can be seen in **Table 2**, participants showed a significant decreasing trend in suicide ideation and attempted one-week after hospitalization (T0) compared to one month (T1) and three months (T2) after discharge. The direction of psychological distress only significantly declined after admission (T0) to one month of discharge (T1), and no significant reduction was found compared with T0 and T2. Further, a significant upward trend can be seen in the quality of life (EQ-VAS), and community integration (CIQ-R) compared to T0 and T1, T2. However, psychological distress, suicide ideation and attempt increased at the 3-month interview (T2) compared with measures at T1. Quality of life and community integration slightly increased from T1 to T2. Even though the changes from T1 to T2 were found non-significantly in our analyses, they indicated a potential rebound in psychiatric symptoms as manifested in depressive symptoms. Notably, after a 3-month discharge, nearly half of the participants revealed recent suicide ideation (46%). Three cases (5.77%) and five (10%) attempted suicide after 1-month and 3-month discharge, respectively. Among eight cases with suicide attempt, one patient attempted twice, so the cumulative incidence of suicide attempts was seven cases (13.46%) in our observation.

**Table 2:** Differences between three-wave interviews regarding suicide ideation and attempt, psychological distress, quality of life, and community integration.

Measures n(%)/ Mean ± SD	Min – Max no/yes	T0 (N = 53)	T1 (N = 52)	T2 (N = 51)	Within subjects effects (F)	R^2^	Between times
Suicide ideation (past 1 week)	0/1	43 (81.10)	20 (38.46)	24 (47.05)	17.85***	0.62	T0>T1***, T0>T2***
Suicide attempt (past 1 month)	0/1	14 (26.42)	3 (5.77)^†^	5 (10.00)^†^	8.21***	0.50	T0>T1***, T0>T2**
BSRS-5	0 – 20	10.69 ± 3.98	8.23 ± 5.67	8.94 ± 5.85	3.35*	0.61	T0>T1*
EQ-VAS	0 – 100	46.45 ± 27.33	64.13 ± 21.96	65.20 ± 22.90	14.73***	0.62	T0<T1***, T0<T2***
CIQ-R	0 – 35	8.34 ± 6.11	13.42 ± 7.23	13.96 ± 7.01	19.87***	0.70	T0<T1***, T0<T2***

*p <.05, **p <.01, ***p <.001; Data are presented as mean ± standard deviation or n (%); ^†^the same patient with twice suicide attempted; BSRS-5, Psychological distress; EQ-VAS, Quality of life; CIQ-R, Community integration; T0, 1 week after hospitalization; T1, 1 month after discharge; T2, 3month after discharge; one-way repeated measure ANOVA in the form of fixed-effect regression model was used to identify trajectory in three-wave interviews.

**Table 3** shows the univariable associations between psychological distress, quality of life, and community integration with suicide ideation and attempt during follow-up. In the bivariate adjusted model, increasing psychological distress was significantly associated with an elevated risk for suicide ideation and suicide attempt with a hazard ratio of 1.11 (95% CI = 1.08 – 1.16; p < 0.001) and hazard ratio of 1.12 (95% CI = 1.03 – 1.21; p < 0.01). Poor quality of life significantly increased the risk by 1.02 (1/0.98) times (B = – 0.02; HR = 0.98; 95% CI = 0.96 – 0.98; p < 0.001) and 1.01 (1/0.99) times (B = – 0.02; HR = 0.99; 95% CI = 0.97 – 1.00; p < 0.05) for suicide ideation and suicide attempt, respectively. In addition, low community integration performance was also a significant predictor for both suicide ideation and attempt with hazard ratios of 1.10 times (1/0.91) (B = – 0.10; HR = 0.91; 95% CI = 0.88 – 0.94; p < 0.001) and 1.06 times (1/0.94) (B = – 0.06; HR = 0.94; 95% CI = 0.88 – 1.00; p < 0.05), respectively.

**Table 3:** Univariable Cox regression analyses predicting of psychological distress, quality of life and community integration associated with suicide ideation and attempt across three-wave interviews.

Predictors	Suicide ideation (past 1 week)		Suicide attempt (past 1 month)	
	B (SE)	HR (95% CI)	B (SE)	HR (95% CI)
BSRS-5	0.11 (0.02)	1.12 (1.08 – 1.16)***	0.11 (0.04)	1.12 (1.03 – 1.21)**
EQ-VAS	– 0.02 (0.01)	0.98 (0.96 – 0.98)***	– 0.02 (0.01)	0.99 (0.97 – 1.00)*
CIQ-R	– 0.10 (0.02)	0.91 (0.88 – 0.94)***	– 0.06 (0.03)	0.94 (0.88 – 1.00)*

*p <.05, **p <.01, ***p <.001; BSRS-5, Psychological distress; EQ-VAS, Quality of life; CIQ-R, Community integration; HR, Hazard Ratio; CI, Confidence Interval.

**Table 4** presents the stepwise multivariate Cox regression model to find the associations between all adjusted baseline characteristics and key variables with suicide ideation and attempt during follow-up. It was found that poor quality of life and low community integration level were likely to increase risks for suicide ideation by 1.02 times (B = – 0.02; HR = 0.98; 95% CI = 0.98 – 1.00; p <0.01) and 1.10 times (B = – 0.09; HR = 0.91; 95% CI 0.87 – 0.96; p < 0.001) for suicide attempt, respectively. In other words, TRD patients with poor quality of life and low community integration level would elevate suicide ideation slightly more than their counterparts. Further, higher psychological distress was the only key variable to predict a higher risk for suicide attempt, with a hazard ratio of 1.13 (95% CI = 1.05 – 1.22; p < 0.001). That means each score higher in psychological distress would increase the risk of suicide attempt 1.13-fold. In addition to the risk related to the baseline demographic, patients who were married or lived with a partner decreased 2.56-fold hazard for suicide attempts (B = – 0.94; HR = 0.39; 95%CI = 0.17 – 0.91; p < 0.05) compared to those who stayed single, divorced, separate, or widowed. Notably, patients who have ever attempted suicide or self-harmed at baseline had the highest risk of suicide attempts in our study (HR = 9.55; 95% CI = 2.81 – 32.46; p < 0.001).

**Table 4:** Multivariate Cox regression model predicting factors associated with the risk of suicide ideation and attempt across three-wave interviews.

Predictors	Suicide ideation (past 1 week)		Suicide attempt (past 1 month)	
	B (SE)	HR (95% CI)	B (SE)	HR (95% CI)
Relationship status (Single^†^ vs Married^‡^)			– 0.94 (0.43)	0.39 (0.17 – 0.91)*
Lifetime suicide attempt/self-harm (No vs Yes)			2.26 (0.63)	9.55 (2.81 – 32.46)***
BSRS-5			0.12 (0.04)	1.13 (1.05 – 1.22)***
EQ-VAS	– 0.02 (0.01)	0.98 (0.97 – 0.99)**		
CIQ-R	– 0.09 (0.02)	0.91 (0.87 – 0.96)***		

*p <.05, **p <.01, ***p <.001; Multivariate Cox regression (stepwise forward method); ^†^Single/divorce/separate/widow, ^‡^Married/lived with partner; BSRS-5, Psychological distress; EQ-VAS, Quality of life; CIQ-R, Community integration; HR, Hazard Ratio; CI, Confidence Interval.

## 4 Discussion

To the best of the authors’ knowledge, the present study is the first to investigate suicide risk associates from inpatient to community follow-ups among patients with TRD. The current study identified a group of clinical TRD patients from one leading national hospital through a rigorous procedure and followed up for three months after discharge. In the Cox regression model, staying single and having a history of suicide attempt were the only baseline characteristics that increased the risk of suicide attempts by 2.56-fold and 9.55-fold in our observation. While the suicide ideation and attempt rates reduced after one-week of hospitalization, the increasing rates at T2 implied alarm signals about recent suicide ideation (46%) and suicide attempts (13.46%) during the 3-month interview period. TRD patients with poor quality of life and low level of community integration performance would elevate suicide ideation slightly more than their counterparts by 1.02-fold and 1.10-fold, respectively. Lastly, a high level of psychological distress also increased the risk of suicide attempts by 1.13 folds in our study.

We identified the high rates of lifetime suicide ideation (98.1%) and lifetime suicide attempt (45.3%) among TRD patients, which percentage were similar compared to a recent finding from Taiwan (94.4% lifetime suicide ideation, 56% lifetime suicide attempt) (20). In our observation, 46% of participants revealed recent suicide ideation, and up to 13.46% attempted suicide which was in line with the escalation of the suicide rate among patients within 3-month after discharge from psychiatric facilities (22, 23, 41). It is worth noting, however, that our observation of suicide ideation and suicide attempt was much higher compared to the findings from the US, with 57.9/100.000 among patients with depressive disorder (22) and 9.3% among TRD patients (41) after discharge three months. Our findings, along with the previous reports, highlight that suicide ideation and suicide attempt within short-term discharge is a global issue regardless of complex general psychopathologic factors (42) and the type of healthcare system in any country. Therefore, any efforts to screen and reduce the suicide rate in TRD patients should be targeted at a broad range rather than any single treatment method. Mental healthcare professionals working with TRD patients should be aware of the elevation of suicide risk after short-term discharge to provide more comprehensive clinical assessment and support to avoid suicide acts.

Although our finding shows the history of suicide attempts at hospital admissions, the hazard of suicide attempts (9.55 times) across the 3-wave interviews was salient and worth attention. However, mental healthcare providers should not overlook TRD patients who did not reveal a history of suicide behaviors because half of the suicides happened without any previous suicide attempt (19, 21, 43). In addition to suicide risk, our results indicated that a higher level of psychological distress was a predictor of suicide attempts by1.13-fold than those with lower psychological distress. This is consistent with the literature, suggesting the predicting role of psychological distress levels (i.e., suicide ideation, depression, inferiority, anxiety, hostility, and insomnia) for eventual suicide during the one-year follow-up (44). On this note, an observation study of claim data from Japan identified that the hazard ratio for completed suicide was 2.37- and 4.16-fold among participants with moderate and severe psychological distress, respectively (27). In particularly, neurobiological factors together with psychological factors play a critical role in severe suicide ideation or suicide attempt and completed suicide (4). Thus, mental health providers should consider that a fluctuating trend of psychological distress from T0 to T2 may lead to a certain risk of suicide among TRD patients in the long-term process. These results suggested that suicide is not only an issue of acute crisis but also a prolonged problem of lasting psychological distress and varies over time, so suicide assessment needs to be repeated and applied to at-risk individuals (44, 45). Therefore, follow-up and regular assessment of patients with TRD after discharge are critically needed, especially for severely depressed patients with suicidal and refractory treatment features (46). This target should be augmented with a greater focus on the safe transition from the hospital to the community. Hence, mental health providers should consider strategies that may improve transition safety, including a pre-discharge safety plan, stress-coping-based psychoeducation, and regular psychological distress assessment for inpatient to outpatient care. Facing limited mental healthcare resources in the Vietnamese community mental health network, telephone follow-up by the mental health nurses with a concise assessment for psychological distress through a short rating scale such as BSRS-5 may be a feasible, effective and reliable way to prevent further suicide.

In addition to suicide risk, our result indicates that lower levels of community integration performance elevated recent suicide ideation among patients with TRD, which was in line with prior finding in Taiwan (20). More importantly, we found a low mean score of community integration in patients with TRD during the follow-up assessments with 8.34 ± 6.11 (T0), 13.42 ± 7.23 (T2), and 13.96 ± 7.01 (T2), which was much lower compared to the norm of the general Australian population (22.33 ± 4.74) (39). These impairments encompass home, social, productivity integration, and electronic social networking, which significantly heighten the risk of recent suicide ideation. Compare to previous work (20), this longitudinal study emphasizes the relationship among total subscale community integration with suicide risk. This is critically important given the unique nature of the chronic illness trajectory in that many participants were on long-term sick leaves and unemployed for several months or years before entering our study. It is also noteworthy early stage of this study was performed during the COVID-19 period, before which Vietnam called coexistence with the pandemic. Hence, routine assessments for people who encounter severe depression could be the first step to improving community integration performance and reducing the incidence of elevated suicide risk under the pandemic stressors.

More specifically, the mean score in quality of life during follow-up period with 46.45 (T0), 64.13 (T1), and 65.20 (T2), which was critical lower than the general Vietnamese adult population with a mean score of 81.08 (36). Consistent with prior research (47), these findings may reflect an unmet treatment need in TRD patients, which is characterized by high disease burden, low quality of life, and reduced function and productivity, with a substantial proportion unable to work. Given our study findings, TRD patients who were not only unable to fulfill multiple medication regimen but, in many cases, worsened in their illness conditions before they were admitted to the hospital. These results and the aforementioned clinical impacts strongly suggest the need to assess TRD patients’ community integration performance to provide a comprehensive approach to reducing personal and societal burdens. The pathways through which community reintegration during the recovery process can influence suicidality among patients with TRD have been identified (20). Enhancing community integration after hospital treatment is of utmost importance for optimal suicide prevention later in TRD patients. Further studies should examine potential intervention strategies to promote adequate four aspects of community integration with randomized controlled trial designs to examine whether improving community integration performance affects not only suicidality but other aspects of TRD long-term outcomes.

Another interesting finding of this study was that the risk of suicide attempts depends on the participants’ marital status at baseline. Those who married or lived with a partner had a lower risk for suicide attempts during the follow-up, with a hazard ratio of 2.56-fold or 61% compared to those who stayed single, divorced, separated, or widowed. This is consistent with the large body of research that has shown that people who have never been married, divorced, or widowed are at an increased risk of suicide compared with those who are married (48). Despite the effectiveness of marital status protection remains controversial, the understandable role has its protective effects through the socio-economic, and emotional support and reduces isolation by providing opportunities for social and community integration (48, 49). The percentage of patients who were married in our study, up to 77.4%, was much higher than 54.4% among the TRD cohort in Taiwan (20). Notably, the lifetime suicide attempt at baseline in our sample was also lower compared to Taiwanese TRD patients with 45.3% and 56%, respectively. Nonetheless, concerning another reason for the effect of marital status on suicide, it remains unknown whether more accurate results would be obtained and control the relevant socio-economic variables or whether combining the same models could produce misleading results. Future studies should examine whether the marital relationship in a more diverse sample of TRD has a clinically significant benefit in suicide and disease modification.

### 4.1 Study strengths and limitations

The strength of our study was its longitudinal design, enabling us to characterize psychological factors, community integration, and suicide over time and claim some causality. Moreover, all the interviews were established and consulted by suicide prevention experts (CYW and MBL) then undertaken by the first author, which could reduce the potential bias. Further, due to a well-established research relationship with the participants, the interviewer could maintain contact with most patients to complete the interview (drop-out rate: n = 2; 3.8%). So, we believe the results were reliable and consistent in our study. Among its possible limitations were the limited sample size and the duration of follow-up observation. Hence, our research team has carefully considered the model and included relevant predictors based on our data and small sample size. In addition, the study participants were recruited from one leading national hospital, which may lead to selection bias and limit the power of generalizability. However, the early stage of data collection was on the severity fluctuation of COVID-19 outbreak condition in Vietnam. Patients could not go to the national hospital for treatment due to the lock-down policy, and the hospital restricted the number of inpatients in the COVID-19 pandemic. Nevertheless, we believe that the level of support perceived by the participants through regular empathetic listening and screening interviews by the researcher should be sufficient for the patients.

## 5 Conclusions

In conclusion, the suicide ideation and suicide attempt rates reduced after inpatient treatment and then altogether increased from T1 to T2 during follow-ups. Our result shows that high psychological distress was associated with a substantial increase of suicide attempt risk. Furthermore, low community integration performance and poor quality of life were slightly correlated with elevated suicide ideation. Lastly, staying single and having a history of suicide attempt were the only baseline characteristics in our observation that predicted a future suicide. These findings suggest for future suicide prevention strategies for individuals with TRD. A regular assessment by healthcare providers from inpatient to outpatient settings using a brief scale such as the BSRS-5 might be useful for early mental distress and suicide risk detection. More importantly, community integration performance should be evaluated sufficiently so that the deterioration of quality of life and suicide ideation can be prevented. Taken together, the potential of these protective factors may ameliorate psychological distress in the otherwise TRD population, with the possibility of lowering the suicide rate in TRD patients during the recovery trajectory in the community.

## Data Availability

The raw data supporting the conclusions of this article will be made available by the authors, without undue reservation.
